# Calciphylaxis in a Biopsy of Pyoderma Gangrenosum in a Patient Without End-Stage Kidney Disease

**DOI:** 10.7759/cureus.31790

**Published:** 2022-11-22

**Authors:** Hassan Mahjabeen, Mark W Kozicky, Elad Kasher

**Affiliations:** 1 Plastic Surgery, St. John's Riverside Hospital, Yonkers, USA; 2 Nephrology, St. John's Riverside Hospital, Yonkers, USA; 3 Internal Medicine Residency Program, St. John's Riverside Hospital, Yonkers, USA

**Keywords:** split thickness skin graft, cutaneous ulcer, non uremic calciphylaxis, calciphylaxis without esrd, idiopathic pyoderma gangrenosum

## Abstract

Calciphylaxis is a rare condition characterized by calcification of the blood vessels in the subcutaneous tissue with tissue ischemia. Calcium deposition is observed within scarred and occluded blood vessels of the subcutaneous tissue when a biopsy of the lesion is performed. Calciphylaxis is usually associated with end-stage kidney disease. We describe the case of a patient with mild renal insufficiency and without end-stage kidney disease, who underwent a wound biopsy with pathology suggestive of pyoderma gangrenosum with calciphylaxis. The wound was successfully treated with surgical debridement, topical antibiotic, and systemic steroid therapy with significant improvement over the course of management. The patient underwent a workup in order to determine the potential causes of calciphylaxis.

## Introduction

Calciphylaxis is characterized by necrosis of the skin and subcutaneous tissue [1,2]. Biopsy typically shows calcium deposition in scarred and blocked blood vessels of the subcutaneous tissue [2]. Calciphylaxis is associated with a variety of disorders, especially end-stage kidney disease [1,2]. Other disease associations include connective tissue disease, obesity, hypoalbuminemia, autoimmune conditions, primary hyperparathyroidism, alcoholic liver disease, diabetes mellitus, and malignancy [1,2]. The use of certain medications such as warfarin, corticosteroids, calcium-based phosphate binders, iron supplements, and activated vitamin D has also been implicated in causing calciphylaxis [1]. Calciphylaxis is an alternative etiology that is considered among the differential diagnosis when evaluating a cutaneous ulcer that appears to be pyoderma gangrenosum [3]. Pyoderma gangrenosum is characterized by full-thickness skin ulceration with purplish undermined borders and is associated with a variety of risk factors including inflammatory bowel disease, rheumatoid arthritis, hepatitis, and granulomatosis with polyangiitis, among others [3,4]. Pyoderma gangrenosum is a clinical diagnosis supported by histology, but it needs to have other, alternative causes excluded as pyoderma gangrenosum is a diagnosis of exclusion [3,4]. A biopsy of the lesion shows a neutrophilic infiltrate, which may be absent on a repeat biopsy as the patient undergoes treatment [3,4]. In this report, we describe the case of an 83-year-old female with a wound suggestive of pyoderma gangrenosum but with calciphylaxis detected in a biopsy of the wound.

## Case presentation

**Figure 1:**
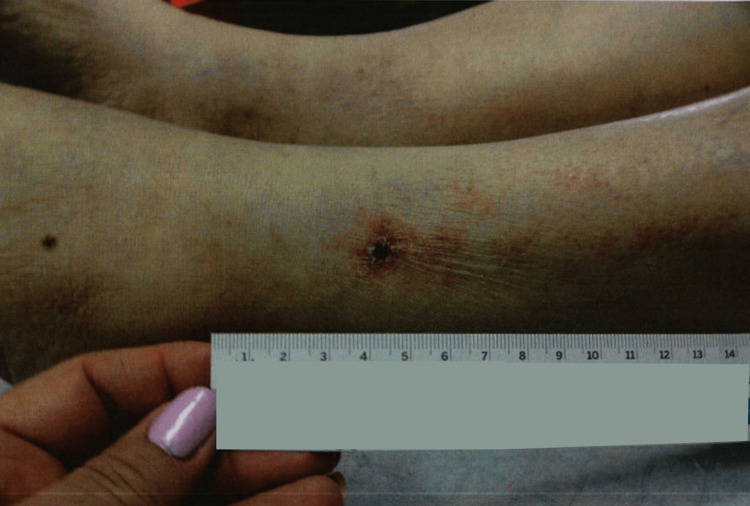
Lower extremity wound initially appearing as a small painful, itchy ulcer

**Figure 2:**
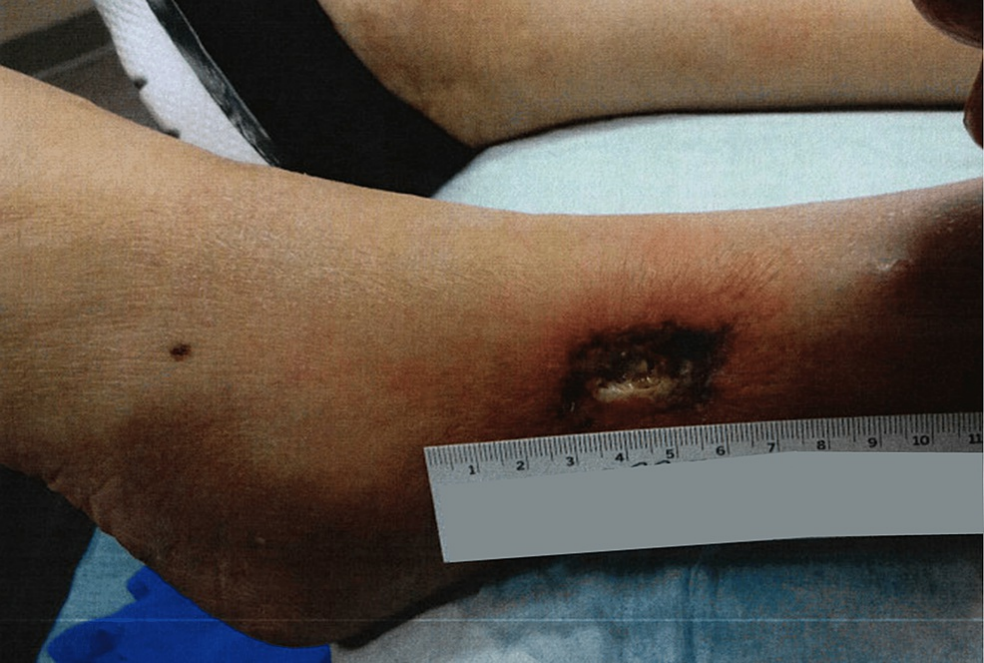
Lower extremity ulceration three weeks after the initial presentation at the wound care clinic

An 83-year-old female presented to the wound care clinic with an ulceration on her left lateral lower leg. Her general medical condition was remarkable for non-insulin dependent diabetes mellitus, hypertension, hyperlipidemia, hypothyroidism due to Graves disease s/p thyroid radiation, recurrent UTIs, lumbar spinal stenosis, fatty liver, and gallstone pancreatitis s/p cholecystectomy. On examination, the patient had a large wound on her left lateral lower leg with significant pain of 10/10 in intensity, as well as clear drainage, and purplish borders around the wound. The wound had initially appeared when the patient had scratched an itch on her lower leg. The area she had scratched had turned into a small painful, itchy ulcer (Figure 1), which had then grown significantly on her left lower leg over the course of several weeks and months with significant pain and clear drainage (Figure 2).

The patient had a workup to identify what kind of ulceration she had. She subsequently underwent debridement of the wound with biopsy, and pathology of the excisional biopsy found inflammation with ulceration and necrosis, as well as acute neutrophilic vasculitis of the small vessels; and calcinosis cutis including dystrophic calcification within necrotic tissue and calcification of small and medium-sized vessels consistent with calciphylaxis. The wound was noted to grow with debridement, suggestive of pathergy. An ultrasound of the lower extremities was only positive for superficial venous insufficiency at the left extremity's mid and distal thigh great saphenous vein. The patient did not exhibit lower extremity edema on physical exam.

Lab work was significant for an elevated proteinase 3 (PR3) level at 6.0 U/mL but was otherwise negative for autoimmune diseases, including a negative ANA, c-ANCA, p-ANCA, SSA/Ro antibody, SSB/La antibody, dsDNA antibody, beta-2 glycoprotein antibody, cardiolipin antibody, phosphatidylserine antibody, anti-prothrombin antibody, and unremarkable C3 and C4 complement levels. The patient also denied any arthralgias or myalgias suggestive of rheumatologic disease, and she also denied any bloody stool or diarrhea indicative of inflammatory bowel disease. An A1C blood test was suggestive of well-controlled diabetes via dietary modifications alone, with the A1C fluctuating between 5.4% and 5.6% during the treatment of the patient's wound. The patient's corrected calcium was within normal limits, fluctuating between 8.4 and 10.1 mg/dL during treatment, with albumin fluctuating between slightly low at 2.9 g/dL and within normal limits at 4.1 g/dL. Similarly, phosphate levels were within normal limits: between 2.6 and 3.5 mg/dL. Her creatinine fluctuated between 0.7 and 1.0 mg/dL with an eGFR between 60 and 85 mL/min/1.73 m^2^, consistent with stage 2 chronic kidney disease. The patient's BMI was in the overweight range, fluctuating between 25.3 and 29.2 kg/m^2^ during the treatment course.

**Figure 3:**
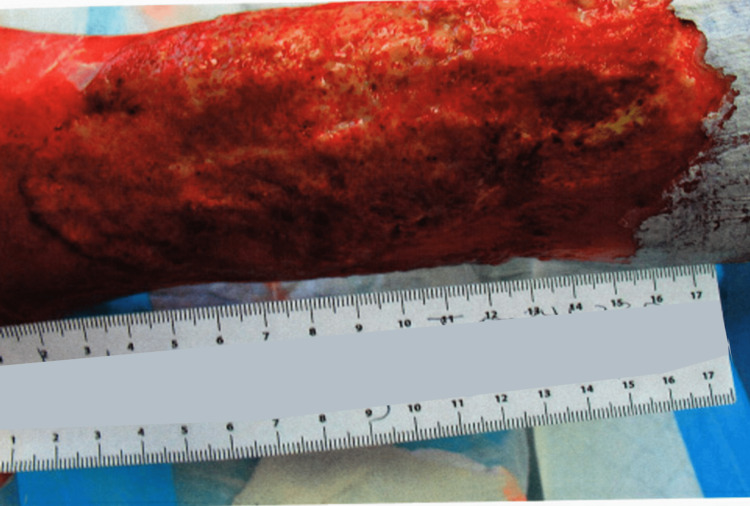
Lower extremity ulceration five months after the failure of two TheraSkin grafts

**Figure 4:**
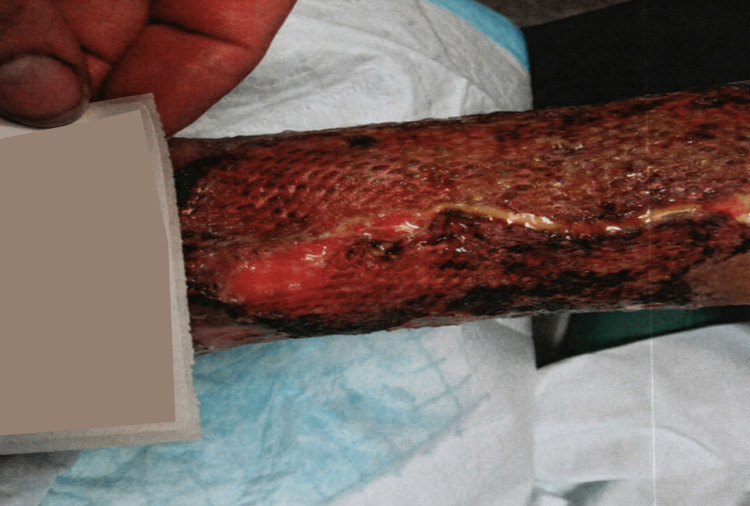
Lower extremity ulceration two weeks after autologous split skin graft application

**Figure 5:**
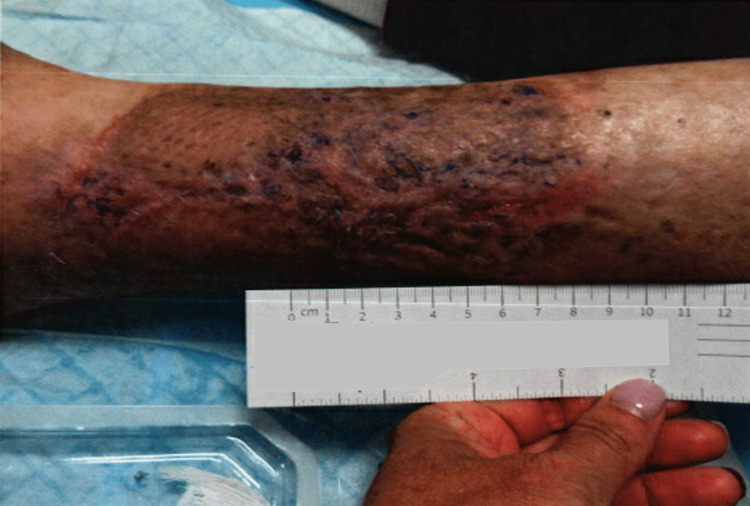
Lower extremity ulceration three and a half months after autologous split skin graft application

The patient was diagnosed as having pyoderma gangrenosum with calciphylaxis and underwent treatment including debridement of the calciphylaxis, application of gentian violet and Iodosorb gel, injection of Celestone steroid with lidocaine as well as oral prednisone, and application of a wound vac, which helped the wound to granulate, use of a compression pump, and hyperbaric oxygen therapy. While debridement of the calciphylaxis did initially cause the pyoderma gangrenosum wound to worsen, the wound healing improved with the use of the wound vac, and an application of TheraSkin was attempted twice, but in vain. However, as the patient progressed in treatment, a repeat biopsy showed continued inflammation but no further evidence of pyoderma gangrenosum and calciphylaxis. The patient’s pain was still a 10/10 in severity after the failed Thera grafts (Figure 3). An application of autologous split skin graft and application of wound vac was later performed, with 80% of the graft being successful (Figure 4). The patient had major improvement in pain reduction with the autologous graft and the pain completely subsided eventually. The autologous graft also led to a significant improvement in the healing of the wound (Figure 5).

## Discussion

This patient's wound biopsy diagnosis of calciphylaxis was an unexpected finding in the pathology report of her wound. Calciphylaxis is associated with a variety of disorders, with moderate to severe chronic kidney disease being the most common one, and is most notably seen in patients with end-stage kidney disease [1]. Other disorders that calciphylaxis is associated with include connective tissue disease, diabetes, obesity, hypoalbuminemia, autoimmune conditions, primary hyperparathyroidism, alcoholic liver disease, diabetes mellitus, and malignancy [1,2]. It is also associated with the use of certain medications such as warfarin, corticosteroids, calcium-based phosphate binders, iron supplements, and activated vitamin D [1].

The patient did not have end-stage kidney disease but only stage two chronic kidney disease with her creatinine fluctuating between 0.7 and 1.0 mg/dL and an eGFR between 60 and 85 mL/min/1.73 m^2^ throughout the course of diagnosis and treatment. Her A1C fluctuated between 5.4% and 5.6% during diagnosis and treatment and was well controlled to be within target goals, and not suggestive of diabetes as a cause of her condition. The patient had a history of fatty liver, and alcoholic liver disease is associated with calciphylaxis, but she reported that her alcohol consumption was limited to an occasional glass of wine at dinner time. The patient's corrected calcium was within normal limits: between 8.4 and 10.1 mg/dL, with albumin fluctuating between slightly low at 2.9 g/dL and within normal limits at 4.1 g/dL, suggesting that she did not have any parathyroid or calcium-associated reason for the calciphylaxis nor hypoalbuminemia as an etiology. Similarly, phosphate levels were within normal limits: between 2.6 and 3.5 mg/dL. The patient's BMI was above 25 kg/m^2^, which placed her only in the overweight range and hence not likely to be the etiology for her calciphylaxis. The patient denied any arthralgias or myalgias to suggest a rheumatologic etiology, but her autoimmune workup was significant for an elevated proteinase 3 (PR3) level at 6.0 U/mL. Also, the patient did have a history of Graves disease, suggesting that her calciphylaxis may have been autoimmune in nature, but the patient's further autoimmune workup under the care of a rheumatologist was otherwise unremarkable. The patient's home medications were also unremarkable and not deemed to be the cause of her condition.

It is possible that the patient’s calciphylaxis was multifactorial, with her elevated proteinase and history of Graves disease, her occasional alcohol use, her mildly elevated BMI, and her stage two chronic kidney disease all contributing to the finding of calciphylaxis on biopsy. The cause of the patient's wound was also unusual. The wound's appearance suggested pyoderma gangrenosum. It was also observed to get worse with debridement, suggestive of pathergy, which is associated with pyoderma gangrenosum lesions [3,4]. Additionally, the biopsy of the wound showed acute neutrophilic vasculitis of the small vessels, which is a biopsy finding associated with pyoderma gangrenosum [3,4]. The neutrophilic infiltrate can disappear on repeat biopsies of pyoderma gangrenosum during treatment; and, in this patient's case also, a repeat biopsy of the wound later in the treatment course did not show a neutrophilic infiltrate [4]. An ultrasound of the lower extremities did find superficial venous insufficiency at the left extremity's mid and distal thigh great saphenous vein. Venous stasis ulcers are associated with findings of dystrophic calcinosis cutis on biopsy, which the patient had; however, the patient did not exhibit lower extremity edema on presentation, which ruled out venous stasis ulcer [5]. The patient's autoimmune workup also showed elevated proteinase 3 levels, suggesting an autoimmune etiology for her wound. However, her autoimmune workup was otherwise negative under the care of a rheumatologist, and the patient did not have any arthralgias, myalgias, or bowel or bladder problems that would suggest an autoimmune etiology or inflammatory bowel disease. Pyoderma gangrenosum is therefore the most likely diagnosis of the patient’s wound.

## Conclusions

Calciphylaxis is a rare condition associated with a variety of disorders. However, the identification of the etiology of a patient’s calciphylaxis may occasionally require an additional workup. The etiology of this patient's calciphylaxis may have been multifactorial, involving her stage two chronic kidney disease, mildly elevated BMI, history of occasional alcohol use, and possibly the elevated proteinase 3 serum level; but no obvious significant risk factor was determined otherwise. The calciphylaxis was successfully treated with a combination of debridement of necrotic tissue, hyperbaric oxygen therapy, and gentian violet, with repeat biopsy showing the resolution of the calciphylaxis. The patient's pyoderma gangrenosum was also successively managed with steroid injections, oral steroids, Iodosorb gel, the use of a wound vac and Tubigrip compression, and the application of an autologous skin graft.
